# Quality of family planning services and associated factors among reproductive age women attending family planning unit at public health facilities in Dire Dawa, Eastern Ethiopia, 2021

**DOI:** 10.1186/s40834-023-00231-1

**Published:** 2023-05-23

**Authors:** Legesse Abera, Ezira Ejigu, Mickiale Hailu, Daniel Tadesse, Abdu Omer

**Affiliations:** 1 Department of Public Health, College of Medicine and Health Sciences in Dire Dawa University, Dire Dawa, Ethiopia; 2 Department of Anesthesia College of Medicine and Health Sciences in Dire, Dawa University, Dire Dawa, Ethiopia; 3 Department of Midwifery College of Medicine and Health Sciences in Dire, Dawa University, Dire Dawa, Ethiopia

**Keywords:** Quality, Family planning, Client satisfaction, Dire Dawa, Health facility

## Abstract

**Introduction:**

Improving the quality of care has been a necessary goal for family planning programs worldwide. Even though extensive work has been done, the contraceptive prevalence rate is still low (41% in Ethiopia, 30.5% in Dire Dawa) and the unmet need for contraception is high (26%) in Ethiopia. Moreover, quality of care in family planning services has an important role in increasing coverage of services and program sustainability. Therefore, the objective of this study was to assess quality of family planning services and associated factors among reproductive age women attending family planning unit in public health facilities in Dire Dawa, Eastern Ethiopia.

**Methods:**

A facility-based cross-sectional study was conducted among reproductive-age women attending a family planning unit in Dire Dawa, Eastern Ethiopia, from September 1–30/2021. A total of 576 clients were selected by systematic random sampling and interviewed using a pre-tested structured questionnaire. SPSS version 24 was used to analyze the data, which included descriptive statistics, bi-variable and multivariable logistic regression analyses. To determine the presence of an association between dependent and independent variables, AOR, P-value 0.05, and 95% CI were used.

**Results:**

A total of 576 clients participated in the study and provided a response rate of 99%. The overall satisfaction of clients with FP services was 79%[95% CI:75.2%, 82.9%]. Having primary education (AOR = 2.11, 95% CI(1.11–4.24), convenient facility opening hours (AOR = 3.13, 95% CI (2.12–5.75), maintaining privacy (AOR = 4.1, 95% CI(2.50–8.12), demonstrating how to use F/P method (AOR = 1.98, 95% CI (1.01–5.20), and discussing F/P issues with husbands (AOR = 5.05, 95% CI: 3.33–7.64) were positively significantly associated with client satisfaction.

**Conclusion and recommendation:**

This study revealed that about four-fifth of the clients was satisfied with the service they received. Clients’ education, facility opening hour, maintained privacy, discussion with husband, and demonstration of how to use the methods were associated with client satisfaction. Therefore, health facility heads should improve facility opening hour. Health care providers should maintain client privacy every time, and should consistently utilize information, education, and communication materials during consultation sessions by giving more attention to client who has no education. Partner’s discussion on family planning issues should also be encouraged.

## Introduction

Family planning is the ability of an individual or couple to decide when to have children, how many children they desire in a family and how to space their children. It is a means of promoting the health of women and families [1]. Family planning services are unique in providing the means for couples to space or limit their births and stabilize the world's population [2].

Studies indicate that maternal, infant and child mortality rates are high wherever fertility is high. In Ethiopia, the maternal mortality rate estimates to be 412 per 100,000 live births, with more than 50% resulting from unsafe abortion, thus making Ethiopian women at the highest reproductive risk in the world [3]. The Ethiopian population policy, adopted in early 1993, aims to reduce the total fertility rate, reduce morbidity and mortality, and raise the contraceptive prevalence rate to a national average of 44% by 2015 [4].

On the other hand, poor quality of care and distrust and alienation created by differences in age, gender, perceived competency and hostility of providers were responsible for many women switching services or stopping use of family planning services entirely. Provision of quality Family Planning service has become the intervention of choice to slow the demographic explosions and part of strategies to reduce the high maternal and child mortality rates. Evidence also showed that low-quality Family Planning service being provided at service delivery points contributed to lessened service utilization [5].

Improving the quality of Family Planning services offers many benefits; information and service will be accessible, clients will make informed decisions, and the public will have a more positive view of health care and its providers. Thus, it is hypothesized to decrease fertility (unplanned population growth) mainly by increasing acceptance, continuation, and hence the prevalence of contraceptive use [6, 7].

Research shows that good quality offers practical benefits to Family Planning clients and programs. These include: -Safety and effectiveness: -Good qualities make contraception safer and more effective. If poorly delivered, some family planning services can cause infection, injuries, and in rare cases, death. Poor services also can lead to incorrect, inconsistent, or discontinued contraceptive use [8].

In this study, the Donabedian model was used**.** Avian Donabedian defined quality of care as “the application of medical science and technology in a manner that maximizes the benefits to health without correspondingly increasing the risk” [9]. His model was developed considering quality of care could involve several formulations depending on where the healthcare system is located. It was intended to assess quality of care in various health services including Family Planning. He identified quality of care as a linear model comprising three components — structure, process, and outcome.

The structure dimension includes all factors affecting the conditions of care-giving such as budget, staff training, reward systems, payment methods, facilities and equipment. The process dimension focuses on what is happening “inside the door” where the provider communicates with the client. The last component of quality is the outcome following provider and client interaction in the clinic. These three parts are interlinked as good structure increases the likelihood of good process, and good process increases the likelihood of a good outcome. Therefore, since client satisfaction considered as one of the desired outcomes of health care, we have used client satisfaction as the outcome variable in this study [9, 10].

Even though different stakeholders have done other work, like increasing the number of health institutions and trained health workers so far, detailed information on the quality of family planning services remains surprisingly limited. In the absence of detailed information on the quality of services, policy discussions on this issue have remained general and concrete recommendations for improving services elusive [11]. Therefore, this study aims to assess the quality of Family Planning services in all dimensions and explore factors affecting the quality of family planning services in Dire Dawa Administration, Eastern Ethiopia. This will help provide evidence-based data to improve and increase Family Planning services quality, utilization and coverage, which in turn improve maternal and child health.

## Methods

### Study setting and design

A facility-based cross-sectional study was conducted from September 1–30/2021, in Dire Dawa, Eastern Ethiopia. Dire Dawa is one of the known and ancient cities in Eastern Ethiopia, which is found around 515 km far from Addis Ababa. Somali Regional State borders it in the east, west and north, and the Oromia Regional State in the south and east. Dire Dawa has a total area of 1,558.64 square kilometers with an estimated density of 237.2 people per square kilometer. The administration's total population of 453,000 in 2016 comprised 227,000 males, 226,000 females and 94,187 childbearing-age women. The total fertility rate of the administration is 3.4 child/ woman and annual population growth rate of 2.9%.

The majority of the population (68%) is urban dwellers [12]. The potential health service coverage of Dire Dawa was 100%, with two governmental hospitals, 15 health centers, and 34 health posts, all providing Family Planning services [13].

## Study population and sampling procedure

The study population includes women of the reproductive age group who visited the family planning services unit at selected public health facilities (2 hospitals and 6 health centers) in Dire Dawa Administration in 2021.

Overall, we recruited 582 family planning users for exit interviews. A standard single population proportion formula was used to calculate the sample size with the assumption of Z^2^α/2 = is (1.96 to be 95% confident level), P = 78% of family planning users satisfied [14], d = is margin of error to be tolerated and taken as 5%, 10% non-response rate and design effect 2. A total of 160 consultation sessions were observed using an observation checklist, and eight public health facilities were checked for logistics related to family planning services.

Stratified and multistage sampling techniques were employed. Public health facilities in Dire Dawa were identified as rural and urban. Then, using simple random sampling, eight health facilities (4 urban and 4 rural Kebele’s) were selected. Again, the calculated sample size was proportionally allocated to each health facility chosen according to the number of client flows from the previous year of a similar month. Finally, study subjects were selected for the study by systematic random sampling technique (k = N/n, 18,080/12 = 1506/582 = 2.6 = 3). The total number of client flow in the selected health facilities was summed up and divided by the sample size required.

## Outcome variable

The dimension employed to measure quality Family Planning in this study was the last component of quality which is the outcome which was measured by satisfaction following provider and client interaction. The main variables under the utilized dimension (outcome) were satisfied and not satisfied.

The theoretical domains behind employing this dimension is that: The quality of care provided in Family Planning service delivery can impact Family Planning use in two ways. Firstly, when clients receive a good quality of care at their first visit, they are more likely to remain in the Family Planning program [15]. Secondly, good quality of care can also result in positive outcomes as clients' satisfaction [15, 16].

## Measurement

### Client satisfaction

This is clients’ opinion of care received from Family Planning services provider and is acknowledged as an outcome indicator of quality of care/service.

- ✓ During analysis the five Likert scale re-categorized/dichotomized as agree and disagree.
- ✓ Clients were satisfied if they agree in greater or equal to the mean score of the items (there were 15 items).
- ✓ Clients were not satisfied if they disagree in greater than the mean score of the items (there were 15 items) [14, 17].

## Data collection tool and procedures

A pre-tested structured interview-administered questionnaire was partly adapted from the literature and some wording or phrasing utilized was modified according to the study's objectives. The questionnaire was first prepared in English and then translated to Amharic and Somali languages, and then back translation was made to see the consistency of the questionnaires. The collected data were also cross-checked daily for its consistency and completeness. Data quality was assured by giving training and appropriate supervision for data collectors. The principal investigator carried out the overall management.

## Data processing and analysis

The collected data were cleaned, edited, coded, and entered into Epi Data version 3.1 and exported to SPSS version 24 for analysis. Descriptive statistics such as frequencies, proportions, and summary statistics were used to describe the study population with relevant variables. Multicollinearity was checked between each independent variable by using Variance Inflation Factors (VIF) to control possible confounding factors. Both bi-variable and multivariable logistic regression analyses were computed. First, bivariate logistic regression analysis was used to assess the presence of association between dependent and independent variables. Then, variables with p-value < 0.25 were screened for multivariable logistic regression and final model fitness was checked by Hosmer–Lemeshow goodness of fit test. Adjusted odds ratios, P-value < 0.05 with 95% CI were used to declare the significance and level of association between the dependent and independent variables.

## Results

### Socio-demographic characteristics of respondents

A total of 576 clients of family planning users were successfully interviewed immediately after receiving care in eight (8) public health facilities and gave a response rate of 99%. As shown in Table 1, 378 (65.6%) were repeat clients, while 198 (34.4%) were new clients. The majority of participants, 253 (43.9%) were between 20- 24 years old with a mean age of 25 years (SD + 4.5). (Table 1).

**Table 1:** Socio-demographic characteristics of respondents at Public Health Facilities in Dire Dawa, Eastern Ethiopia, 2021

**Variables**		**No**	**%**
Respondents age group	15–19	96	16.7
	20–24	253	43.9
	25 and more	227	39.4
Types of Clients	Repeat	378	65.6%
	New	198	34.4%
Religion	Orthodox	208	36.1
	Muslim	236	41.0
	Protestant	84	14.6
	Catholic	48	8.3
Ethnicity	Oromo	341	59.2
	Somali	208	36.1
	Amhara	27	4.7
Place of Residence	Urban	392	68.1
	Rural	184	31.9
Educational status	Not read and write	222	38.1
	Primary school	203	36.0
	Secondary school & above	151	25.9
Occupation	Housewife	270	46.9
	Employee	116	20.1
	Merchant	96	16.7
	Daily labourer	94	12.4
Marital status	Not married (single)	24	4.2
	Married & live together	512	88.9
	Widowed &divorced	40	6.9
Have you ever discussed about	Yes	512	88.9
F/P with your husband/partner?	No	64	11.1

### Availability and functionality of logistic supplies for family planning services

Eight family planning service delivery points (health facilities) were audited for the availability and functionality of family planning services logistics, and supplies that were crucial to function effectively and could affect the quality of care provided.

### Satisfactory facility environment

Among eight health facilities, only five had a clean water supply, and 50% had toilets with poor sanitation. In three of the health facilities, there was a shortage of staff assigned to work in family planning services and in all health facilities there was a shortage of trained providers. All health facilities' official working days and hours were from Monday to Friday from 8:30 am to 5:30 pm except for lunch time (12:30 am-8:00 pm). All health facilities have an examination couch and clients seating and offer visual privacy but not auditory privacy during a pelvic examination, IUCD insertion or during consultation time. Based on the findings, the facility environment in our study area was satisfactory.

### Availability of minimum equipment to offer Family Planning services

The result showed blood pressure measurement apparatus, weight scale, and stethoscope were available in all health facilities, but stethoscopes were shared with other departments in 3 (37.5%) of health facilities. Uterine sound, tenaculum, speculum, and scissors were unavailable in 2 (25%) facilities. All health facilities had essential disposable items like needles, syringes and gloves. All health facilities had laboratory units, but 2 (25.0%) of health facilities had not been performing pregnancy tests because of the absence of kits for pregnancy tests. Hence, all health facilities fulfilled the minimum equipment to offer family planning services.

### Availability of Family Planning contraceptive supplies

The assessment result indicated that combined pills, progestin-only pills, Depo Provera, Implanon and condoms were available in all setups. However, IUCD was only available in three health facilities. The procedure for tubal legation and vasectomy was carried out in two hospitals.

**Fig. 1:**
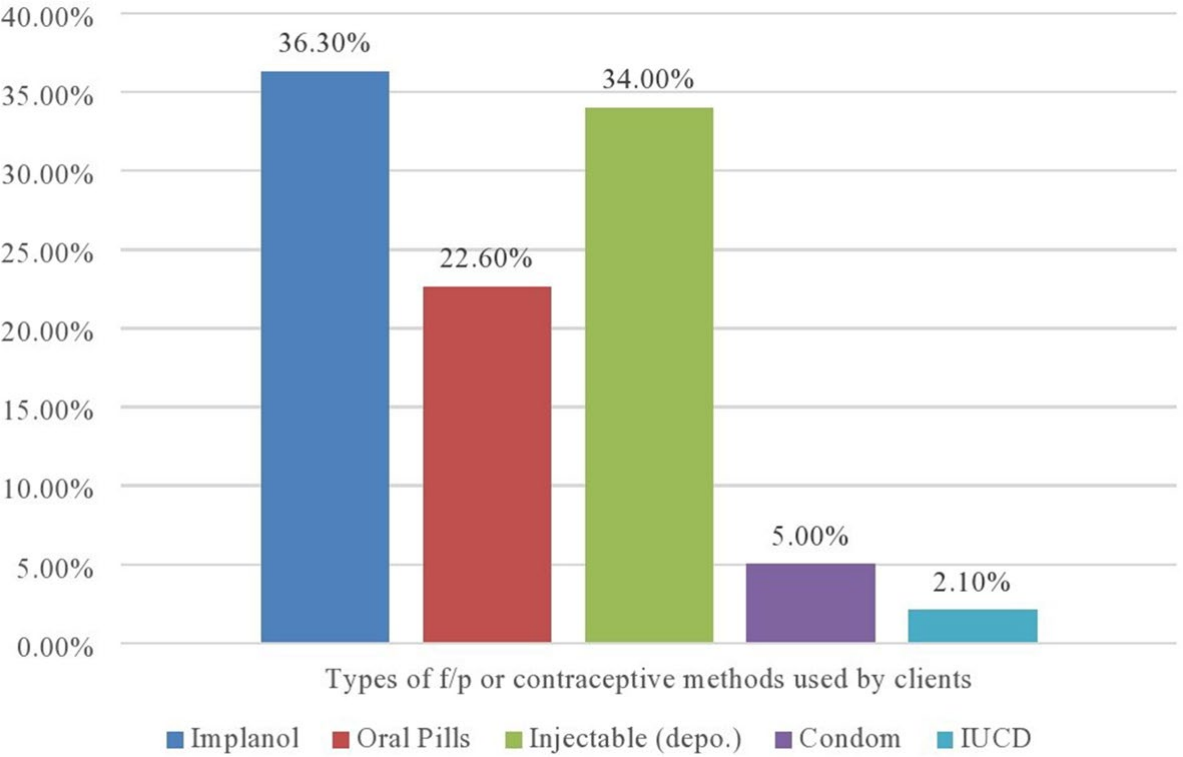
Types of contraceptive methods frequently used by clients in assessing the quality of care in f/p services among public health facilities in Dire Dawa, Eastern Ethiopia, September 2021

As shown in Fig. 1; Client exit interviews showed the majority, 209 (36.3%) of clients were using/received Implanon, 196 (34.0%) of clients were using injectable (Depo-Provera), 130 (22.6%) of clients were using pills, 29 (5.0%) using a condom and only 12 (2.1%) using IUCD (Fig. 1). All of the health facilities had recording systems for received and dispensed FP commodities and adequate storage facilities. Stores were protected from sun, rain, wet, and rats in all health facilities. Based on the above information, the minimum standard of contraceptive supply is achieved.

### Availability of reproductive health guidelines and FP IEC materials

**Fig. 2:**
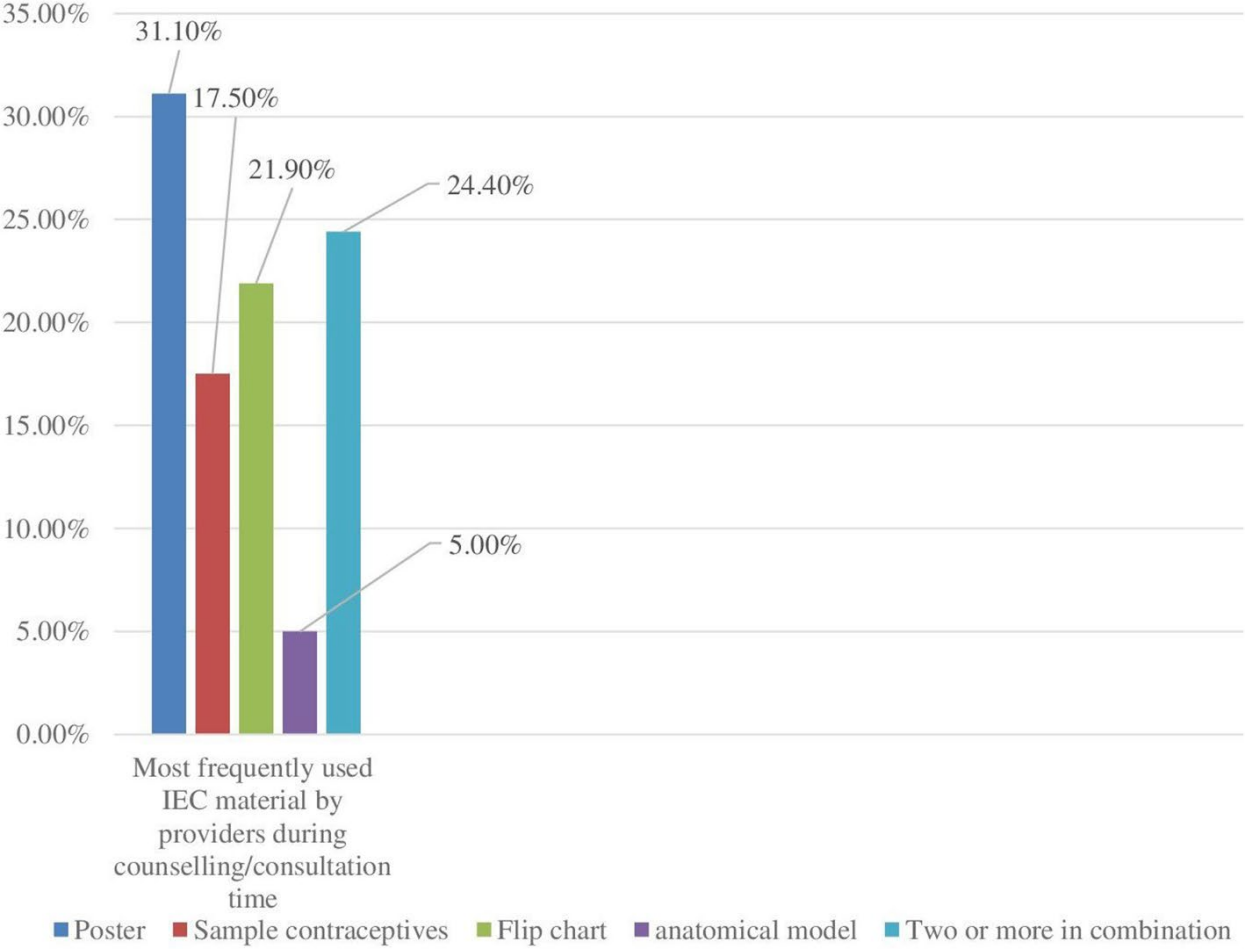
Types of IEC materials frequently used by providers during client counseling/consultation time by observation among public health facilities in Dire Dawa, Eastern Ethiopia, September 2021

Only 4 (50%) health facilities had a copy of Ethiopian Minister of Health (EMOH) guideline of family planning and reproductive health services and achieved the requirement. All health facilities had at least one information education and communication (IEC) material but not three at a time. Regarding IEC materials during consultations, 35 (21.9%) were used flip charts, 28 (17.5%) were displayed samples of contraceptives, brochures were used by 8 (5.0%) of clients and posters were used by 50 (31.3%) of clients. A combination of two or more IEC materials was used for 39 (24.4%) clients (Fig. 2).

### Appropriate constellation of services

By exit interview, the distance of clients’ homes from the service delivery was estimated by the clients themselves and 311 (54.0%) of clients were reported that they traveled less than 30 min, 201 (34.9%) of clients were traveled 30–60 min and 64 (11.1%) of clients were traveled more 60 min. Regarding the opening hours of health facilities, 452 (78.5%) of the clients agreed that the opening hour was convenient for them, and 124 (21.5%) of clients disagreed with opening hours. The median waiting time was 25 min and SD ± 20 min, ranging from 5 to 45 min. The waiting time of clients at the service delivery points before getting services, 503 (87.3%) of clients were reported that the waiting time was short, and 73 (12.7%) of clients were informed that the waiting time was long (Table 2).

**Table 2:** Client’s accessibility to health facility and F/P information

Information told about the preferred method (*n* = 576)		No	%
Variable			
Source of F/P service information for first time	Husband	211	36.6
	Neighbors	150	26.0
	Health professional	215	37.3
Distance of client’s home from health facilities	Less than 30 min	311	54.0
	30–60 min	201	34.9
	More than 60 min	64	11.1
Health facility opening hours convenient?	Yes	452	78.5
	No	124	21.5
Feeling of clients about waiting time	It was short	503	87.3
	Long time	73	12.7
Getting desired information & service	Yes	534	92.7
	No	42	7.3
Will you come back for next appointment?	Yes	534	92.7
	No	42	7.3

### Provider competency for provision of family planning services

As shown in Table 3 above, Four hundred seventy (81.6%) of the respondents reported that the provider explained how the method works. Almost all, 530 (92.0%) of the respondents reported that the provider demonstrated how to use the method, 455 (79.0%) of providers described possible side effects, 464 (80.6%) of providers explained what to do for side effects, 496 (86.1%) of providers told to their clients about the possibility of changing method and 473 (82.1%) of them mentioned when and where to go for supply or follow-up.

**Table 3:** Information given to clients by service provider

Information told about the preferred method (*n* = 576)	Yes (%)	No (%)
Demonstrate how to use method	530 (92.0%)	46 (8%)
Explained how the method works	470 (81.6%)	106 (18.4%)
Described about possible side effects	505 (87.7%)	71 (12.3%)
Explained what to do for side effects	464 (80.6%)	112 (19.4%)
Told about possibility of changing method	496 (86.1%)	80 (13.9%)
Mentioned when and where to go for supply or follow-up	473 (82.1%)	103 (17.9%)

### Client-provider interaction from client perspective

As shown in Table 4; 430 (74.6%) of clients reported that consultation time with the provider was about right (appropriate), 146 (25.4%) of clients were informed that the consultation time was short, and 52 (9.0%) of the clients were did not want to respond. Moreover, 527 (91.5%) of the clients reported that the provider was easily understandable, and 36 (6.3%) of the clients said that the provider was challenging to understand (Table 4).

**Table 4:** View of clients on the process of obtaining service at Public Health Facilities in Dire Dawa, Eastern Ethiopia, 2021

Variable		No	%
Consultation time with service provider	About right	430	74.6
	Short	146	25.4
Provider was easy to understand	Easily understandable	540	93.7
	Difficult to understand	36	6.3
Why you preferred this health facility	I can choose provider	355	61.6
	Provide quality services	115	20.0
	Integrate service provided	69	12.0
	Near to my home	37	6.4
Will you come back for next appointment	Yes	511	88.7
	No	65	11.3

### Client satisfaction with family planning services provided

As Table 5 shows, among all Family Planning (FP) users interviewed, 517 (89.8%) were satisfied with FP counseling given by service providers, while 59 (10.2%) of them were not satisfied. The majority of the respondents were satisfied with the ease of getting Family Planning unit (89%).

**Table 5:** Satisfaction of clients on F/P services at Public Health Facilities in Dire Dawa, Eastern Ethiopia, 2021

Attitude questions or statements	Agree (%)	Disagree (%)
Provider greeting is good and in a friendly way	523 (90.8%)	55 (9.2%)
Provider performs the procedure with cleanliness and sanitation	513 (89.1%)	63 (10.9%)
Provider has good knowledge and skill to perform the procedure	466 (80.9%)	110 (19.1%)
Sufficient family planning methods are available	458 (78.7%)	118 (20.5%)
Information given about the method is sufficient/adequate	560 (97.2%)	16 (2.8%)
Waiting time is fair and adequate	503 (87.3%)	73 (12.7%)
Privacy was maintained	536 (93.0%),	40 (7.0%)
Waiting place is adequate with latrine and water supply	445 (77.3%)	131 (22.7%)
Consultation time with provider is appropriate time	378 (65.6%)	198 (34.4%)
During consultation, the provider is easy to understand	527 (91.5%)	49 (8.5%)
Quality f/p services can be better provided by doctor	378 (65.6%)	198 (34.4%)
Quality f/p services can be better provided by female nurse	513 (89.1%)	63 (10.9%)
Quality f/p services can be better provided by male nurse	312 (54.2%)	164 (45.8%)
Quality f/p services can be better provided by HEW	201 (34.9)	375 (65.1%)
Do you agree that you have received quality f/p services from this health facility and satisfied?	455 (79.0%)	121 (21.0%)

Overall satisfaction of clients with family planning services was indicated that, 455 (79%) [95% CI: 75.2%, 82.9%] of the clients were satisfied with the service they received, and 69 (12.0%) of the clients were not satisfied (Table 5).

### Factors associated with client satisfaction with family planning services

In bi-variable logistic regression analyses, client’s residence, educational status, facility opening hours, short waiting time, maintained privacy, having family planning discussion with husbands, sufficient consultation time, demonstration of how to use the methods, and proper explanation of side effects was significantly associated with client satisfaction.

In multivariable logistic regression analysis again, client education, the convenience of facility opening hours, maintained privacy, demonstrated of how to use the method, and having family planning discussions with husbands were significant associations with client satisfaction.

Clients with primary education were 2.11 times more likely satisfied with family planning services provided them than those with secondary education and above (AOR = 2.11, 95%CI (1.11–4.24). Clients who agreed that the facility opening hour was convenient for them were 3.13 times more likely satisfied than those who disagreed with the facility opening hour (AOR = 3.13, 95% CI (2.12–5.75).

Family planning users whose privacy was maintained during examination and procedure were four times more likely satisfied than those whose privacy was not maintained (AOR = 4.1, 95% CI (2.50–8.12). Clients who demonstrated how to use the method were 1.98 times more likely to be satisfied than those who were not demonstrated (AOR = 1.98, 95% CI (1.01–5.20).

Clients who had family planning discussions with husbands were five times more likely satisfied than those who had no family planning discussion (AOR = 5.05, 95% CI: 3.33–7.64) (Table 6).

**Table 6:** Factors associated with client satisfaction with F/P services

Variables	Satisfaction		COR (95%CI)	COR (95%CI)
	Yes	No		
Respondent’s age				
15–24 years	150	199	1.00	1.00
> = 25 years	77	150	0.178(0.12–0.27)	0.390 (0.19–2.541)
Marital status of respondents				
Single	10	14	1.00	1.00
Married*1	364	188	1.591(1.77–3.17)	2.156(0.674–6.89)
Have discussion with husband on F/P				
No	18	46	1.00	1.00
Yes	267	245	4.429(3.15–5.28)	5.05 (3.33–7.64) **
Having children				
No	18	22	1.00	1.00
Yes	266	270	0.91(0.23–3.55)	2.71(0.99–6.01)
Breast-feeding				
No	106	160	1.00	1.00
Yes	120	150	1.13 (2.12–5.75)	1.98 (0.62–4.35)
Educational status				
Secondary school and above	71	80	1.00	1.00
Primary education and less	223	202	1.20 (1.32–6.15)	2.11 (1.11–4.24) **
Convenient opening hours of facility				
No	64	60	1.00	1.00
Yes	196	256	2.61(1.13–4.55)	3.13 (2.12–5.75) **
Privacy maintained				
No	11	29	1.00	1.00
Yes	300	236	2.1(1.92–4.32)	4.10 (2.50–8.12) **
Demonstrated how to use the method				
No	11	35	1.00	1.00
Yes	325	205	1.50 (1.11–3.23)	1.98 (1.21–5.20) **

## Discussion

It has been said that good quality family planning service helps individuals and couples to meet their reproductive health needs safely and effectively. Client satisfaction is essential to clients’ decisions to use and continue the service for their future. And it is a core indicator of the quality of service. This study showed that, the overall satisfaction of clients with Family Planning services was 79% [95% CI: 75.2%, 82.9%]. This finding is almost consistent with the studies conducted in Metu Karl Referral Hospital, South West Ethiopia (78%), Hosana (75%), Kenya (75.3%), and Nigeria (81%) [14, 17–19]. The possible reasons for the similarity could be the perceived sufficiency of consultation, and low expectations of clients on Family planning of service at public health facility.

But the finding is higher than the finding of the studies conducted in Wonji Hospital, Ethiopia (42%), and Jijiga (41.7%) [20, 21]. The possible explanation might be socio-demographic differences between the study populations in the above studies.

However, the result of our study is lower than studies conducted in Jimma zone, Southwest Ethiopia (93.7%), Senegal (84%) and Mozambique (85%) [22–24]. The possible reason for this discrepancy could be this study didn’t include the private and non-governmental health facilities compared to the above studies. Hence, the level of client satisfaction is expected to be higher in private health facilities [25].

Studies in the developing world have shown a clear link between patient satisfaction and various explanatory factors, among which service quality has been prominent. The finding of this study revealed that Clients with primary education were 2.11 times more likely to be satisfied than those with secondary education and above. This finding was consistent with a study conducted in Metu Karl Referral Hospital, South West Ethiopia [14]. The possible explanation could be that the more educated the clients, the more they expect quality services than the actual services provided.

The convenience of facility opening hours was a predictor of client satisfaction. Clients who agreed that the facility opening hour was convenient were 3.13 times more likely to be satisfied than those who disagreed with the opening hour. This finding is consistent with a study conducted in Hosanna town health facilities [17]. The possible explanation could be that the opening hour is convenient for clients; they receive the service early and utilize their time properly for other duties, increasing their satisfaction.

In this study, family planning users who were demonstrated how to use family planning of their choice were about two times more likely satisfied with the services compared to those who were not demonstrated. This finding is consistent with the studies conducted in Metu Karl referral Hospital, South West Ethiopia, Hosanna town, and Jimma zone, Southwest Ethiopia [14, 17, 24]. This finding might be related to the fact that the information provided and demonstrated during service contact enables clients to choose and use contraception, increasing their understanding of how to use the methods, thereby increasing client satisfaction. Clients' lack of information on how to use it results in a negative attitude towards methods whenever they experience certain side effects. This might lead to dissatisfaction and the client might discontinue the chosen family planning method.

This study again revealed that Family planning users whose privacy was maintained during examination and procedure were four times more likely satisfied than those whose privacy was not maintained. This finding is in line with the study conducted in Hosanna town [17]. The possible explanation could be privacy is very important culturally and they consider it as the provider respecting their dignity, which increases their satisfaction.

Studies showed that having a family planning service discussion with the husband is a principal factor leading to a high rate of satisfaction and program and method continuation [26]. In this study, clients who had family planning discussions with husbands were five times more likely satisfied than those who did not discuss with their husbands. This result was similar to the Jimma studies [27]. This could be because as a free discussion between partners, women can easily meet their needs and free talk with providers, enhancing satisfaction.

Long waiting time is a principal factor leading to a high rate of program and method discontinuation [26]. In this study, 87.3% of clients agreed that the waiting time was acceptable (short), which was a similar finding to the studies conducted in Bangladesh (71.8%) of clients were satisfied [28]. This is far higher than the USAID analysis on the quality of family planning report from Ghana, Kenya, and Tanzania, in which 42.1% and 69% of clients got service within the acceptable waiting time, respectively [25]. But lower than the study in Jimma (92.4%) [27]. This variation could be because of the difference in facilities working culture, client flow and the recent reform implementation in the current study setting.

Welcoming or greeting clients in the first contact at service delivery points enhances the interaction as it has emotional contents of exchange between provider and clients. In this study, 130 (81.3%) clients were provided a warmly greeted in a respectful and friendly way by their care providers. This result is better than the study conducted in Jimma (65.3%) of the providers welcomed their clients [24]. This indicates most of the family planning providers are not consistently working as per the guideline, which compromises the quality of FP services. This might be due to provider neglect, shortage of time, or client overflow.

Privacy is one of the main criteria for assessing care quality [29]. In our study, 536 (93.0%) of the clients responded that their privacy was maintained, but in observation, privacy was maintained in (78.1%) of clients. This study result is better than the study conducted in northwest Ethiopia (33.7%) [30]. But in our study, similar to the above study, auditory privacy was not maintained in around three-fourths of observed clients. This low auditory privacy could result from a lack of separate room for FP services.

## Conclusion and recommendation

In conclusion, quality is rapidly becoming a global issue and concern to both the provider and the users of health services. This study identified that about four-fifth of the clients were satisfied with the service they received. Having education, convenience of facility opening hours, maintaining privacy during examination and procedure, demonstrating how to use FP methods were positively associated with client satisfaction with family planning service. Therefore, health facility heads should improve facility opening hour. Health care providers should maintain client privacy every time, and should strengthen the utilization of IEC materials during consultation and demonstration sessions by giving more attention to client who has no education. Partner’s discussion on family planning issues should also be encouraged.

## Data Availability

Data related to this manuscript is available on the hand of corresponding author and will be obtained under a reasonable request.
